# Single cell RNA sequencing reveals ferritin as a key mediator of autoimmune pre-disposition in a mouse model of systemic lupus erythematosus

**DOI:** 10.1038/s41598-021-03649-2

**Published:** 2021-12-20

**Authors:** Subhi Talal Younes, Kurt Showmaker, Ashley C. Johnson, Michael R. Garrett, Michael J. Ryan

**Affiliations:** 1 Department of Pediatrics, Boston Children’s Hospital, Boston, MA USA; 2 Department of Pharmacology and Experimental Therapeutics, University of Mississippi Medical Center, Jackson, MS USA; 3 Department of Pharmacology, Physiology, and Neuroscience, University of South Carolina School of Medicine, 6439 Garners Ferry Road, Columbia, SC 29209 USA

**Keywords:** Autoimmunity, Systemic lupus erythematosus

## Abstract

Systemic lupus erythematosus (SLE) is a devastating autoimmune disorder characterized by failure of self-tolerance with resultant production of autoreactive antibodies. The etiology of this syndrome is complex, involving perturbations in immune cell signaling and development. The NZBWF1 mouse spontaneously develops a lupus-like syndrome and has been widely used as a model of SLE for over 60 years. The NZBWF1 model represents the F1 generation of a cross between New Zealand Black (NZB) and New Zealand White (NZW) mice. In order to better understand the factors that contribute to the development of autoimmunity, single cell RNA sequencing was conducted using the bone marrow from female NZBWF1 mice prior to the development of overt disease. The results were contrasted with single cell RNA sequencing results from the two parental strains. The expected findings of B cell abundance and upregulation, and evidence of interferon signaling were validated in this model. In addition, several novel areas of inquiry were identified. Most notably, the data showed a marked upregulation of the ferritin light chain across all cell types in the NZBWF1 mice compared to parental controls. This data can serve as a gene expression atlas of all hematopoietic cells in the NZBWF1 bone marrow prior to the development of autoimmunity.

## Introduction

Systemic lupus erythematosus (SLE) is a multisystem autoimmune syndrome characterized in part by the production of a variety of auto-reactive antibodies^1^. The etiology of this complex syndrome is multi-factorial, involving both genetic susceptibility^2^ and complex environmental factors^3^. Consequently, the study of SLE has leveraged a variety of similarly multi-factorial model systems^4^ including mouse strains with polygenic susceptibility to autoimmunity^5,6^. One such strain, the New Zealand Black-White F1 (NZBWF1), is produced by crossing a female New Zealand White (NZW) and male New Zealand Black (NZB). The resultant female F1 progeny exhibit an SLE-like phenotype characterized by production of anti-double stranded DNA antibodies and nephritis^7^.

Prior studies in the NZBWF1 model show multiple genetic susceptibility loci which collectively contribute to the phenotype^6,8^. However, the precise mechanisms which contribute to the autoimmune predisposition in this strain are not clear. Importantly, the identification of genes that impart susceptibility to autoimmunity in NZBWF1 mice led to the discovery of orthologous genes linked to autoimmunity in humans^9^. Thus, the continued study of underlying genetic factors contributing to SLE in NZBWF1 mice can lead to a better understanding of human SLE. In order to advance this cause, single cell RNA sequencing was conducted using the bone marrow from 9 to 10-week-old female NZBWF1 mice and the two parent strains, NZW and NZB. This age was selected because it precedes the development of overt autoimmunity. The purpose of this study was to define the genetic and cellular landscape of this model’s hematopoietic system prior to the development of any disease phenotype. In so doing, several areas of novel inquiry were identified while simultaneously validating its use as a faithful small animal model of SLE.

## Methods

### Animals

Seven-to-eight-week-old female New Zealand Black (NZB), New Zealand White (NZW), and New Zealand Black-White F1 (NZBWF1) mice (n = 3) were purchased from The Jackson Laboratory, and were delivered at the same time. Animals were maintained on a 12-h day/night cycle and allowed to feed (standard chow) and drink ad libitum until they reached 9–10 weeks of age to conduct the study. Twenty-four hours prior to euthanasia, mice were placed in metabolic cages for collection of urine. At approximately 7:00 am, isoflurane was administered at a high dose until respirations ceased; cervical dislocation was then performed, and tissues were harvested. All studies were reviewed and approved by the University of Mississippi Medical Center Animal Care and Use Committee and comply with ARRIVE guidelines. All methods were carried out in accordance with relevant guidelines and regulations.

### Bone marrow cell collection

The left and right femurs of the mice were disarticulated and dissected away from muscle. Each end of the bone was then clipped. Five milliliters of Hank’s Balanced Salt Solution was flushed through each end of the bone using a 30-gauge needle. This solution was passed through a 70-micron filter to remove large pieces of debris. The cells were spun down at 200 rcf for five minutes and re-suspended in 1 mL of phosphate-buffered saline (PBS) with 0.04% bovine serum albumin. Four milliliters of RBC lysis buffer from BD Biosciences (catalog number 555899) was added and the solution was incubated at room temperature for 5 min away from light. Cells were centrifuged again at 200 rcf for 5 min and resuspended in 1 mL of PBS with 0.04% BSA. The centrifuged cells were washed three times according to the following protocol: centrifuge at 150 rcf for 3 min, remove the supernatant, resuspend the cells with a wide-bore pipette tip in 1 mL of PBS with 0.04% BSA. After the final wash and resuspension, cell concentration and viability was determined using a Bio-Rad TC20 automated cell counter.

### Single cell RNA sequencing

Bone marrow cells were isolated using the 10 × Genomics Chromium Single Cell controller according to the manufacturer’s instructions using the 10 × Chromium Single Cell 3’ v3.1 Reagent Kit (10 × Genomics Product Code 1000268) followed by cell lysis, and library preparation including barcoded RNA reverse transcription, cDNA amplification, addition of sample index, and Illumina P5/P7 adapters. The libraries were sequenced using an Illumina NextSeq 500 instrument. Reads from the NextSeq instrument were initially analyzed using the 10 × Genomics Cell Ranger software v3.1. FastQ files were generated, samples de-multiplexed, and gene counts obtained using the Cell Ranger mkfastq, count, and aggr commands. Resulting output tables were then imported into R statistical software for further analysis.

### Data analysis

Data analysis was performed using R and a variety of Bioconductor packages^10–30^ following many of the conventions described by Amezquita and colleagues in the book *Orchestrating Single Cell Analysis with Bioconductor*^31^. The code associated with this analysis is available on GitHub (https://github.com/styounes/SLE_scRNAseq).

HDF5 (.h5) files for each sample were loaded using the read10xCounts function of the DropletUtils package. Most of the following downstream analyses were performed using a combination of the scran and scater packages. Per cell quality control metrics included total detected unique molecular identifies (UMI), number of expressed genes, and the percentage of mitochondrial genes. Cells with less than 1000 total UMIs, less than 500 unique genes, or greater than 10% expression of mitochondrial genes were removed from the dataset. There was no difference in quality of cells among batches or across strains. These metrics exhibited a bimodal distribution (Supplementary Fig. 1A) with a clear inflection in between low- and high-quality cells, guiding the selection of thresholds. In order to ensure that highly metabolically active cells were not discarded by this approach, the total library size (i.e. total UMIs) was plotted against the percentage of mitochondrial genes for each cell (Supplementary Fig. 1B). Given the fact that there were no cells with large library sizes (presumably high-quality cells) with concomitant large proportion of mitochondrial genes, it was concluded that this approach did not inadvertently discard any metabolically active cell populations. Finally, the enrichment of gene subsets within discarded cells was checked and, observing no such enrichment, demonstrated that quality control was not discarding a distinct population of cells (Supplementary Fig. 1C). Doublets (droplets which contained two or more cells) were identified using a simulated doublet approach whereby artificial doublets are constructed from the dataset and cells which lie close to these artificial doublets in high-dimensional space are removed (guilt by association), as implemented in the scDblFinder function with the following non-default parameters: nfeatures = 750, propRandom = 1.

Gene expression was normalized by pooling counts from related cells and calculating a size factor for each pool; cells were then deconvolved into cell-based size factors for normalization of each cell’s expression profile, using the functions quickCluster and computeSumfactors as implemented in the scran package. Genes with a high variance of expression after applying a correction factor for abundance were selected for use in several downstream analyses, most notably, dimensionality reduction. The top 20% of highly variable genes were selected.

Cell types were annotated using a curated set of marker genes garnered from the Cell Marker database (http://bio-bigdata.hrbmu.edu.cn/CellMarker/) (Supplementary Table 1). Equipped with these gene sets, an assignment score for each permutation of cell and cell type was computed using the function AUCell with the following nondefault parameters: aucMaxRank = 750. Diagnostic plots were inspected and score thresholds manually adjusted for optimal assignment in our dataset.

Differential gene expression among strains was determined by aggregating cell types across samples using the function aggregateAcrossCells implemented in the package edgeR. Samples with less than 10 cells for the given cell type were removed. Differential gene expression was computed by the function pseudoBulkDGE from the scran package. Briefly, this function loops across cell types and performs differential expression using a quasi-likelihood method as implemented in the package edgeR. For the design matrix NZB-NZBWF1-NZW, the coefficients were: − 0.5, 1, − 0.5. Similarly, differential abundance of cell populations was computed using quasi-likelihood method with a design matrix and coefficients as above. Phase of the cell cycle for each cell was inferred based on the expression of stereotypic cell cycle genes using the package cyclone.

### Subclustering

B-cell subclusters were determined as follows. After appropriate subsetting of the global dataset, dimensionality reduction by principal component analysis was repeated. In order to minimize technical noise while maximizing identification of true biological differences, clustering was run using a subset of these principal components in keeping with the method described by Amezquita et al.^30^. The number of components was determined by first computing the number of clusters vs. the number of principal components used in clustering. Next, the number of principal components which yields no more than PC + 1 clusters was selected, as it represents the inflection point between over- and under-clustering. A k-nearest-neighbor approach was applied to the dataset as implemented in the function buildSNNGraph in the igraph package (Supplementary Fig. 2A). Cluster stability was evaluated based on both cluster modularity and bootstrapping approaches (Supplementary Fig. 2B,C). One subcluster (label 1) was removed as this subcluster had a high library size and expressed both myeloid and lymphoid markers, suggesting it consisted mostly of doublets. Differential abundance and expression across B-cell subclusters was determined similar to that described above for other cell types.

## Results

**Figure 1:**
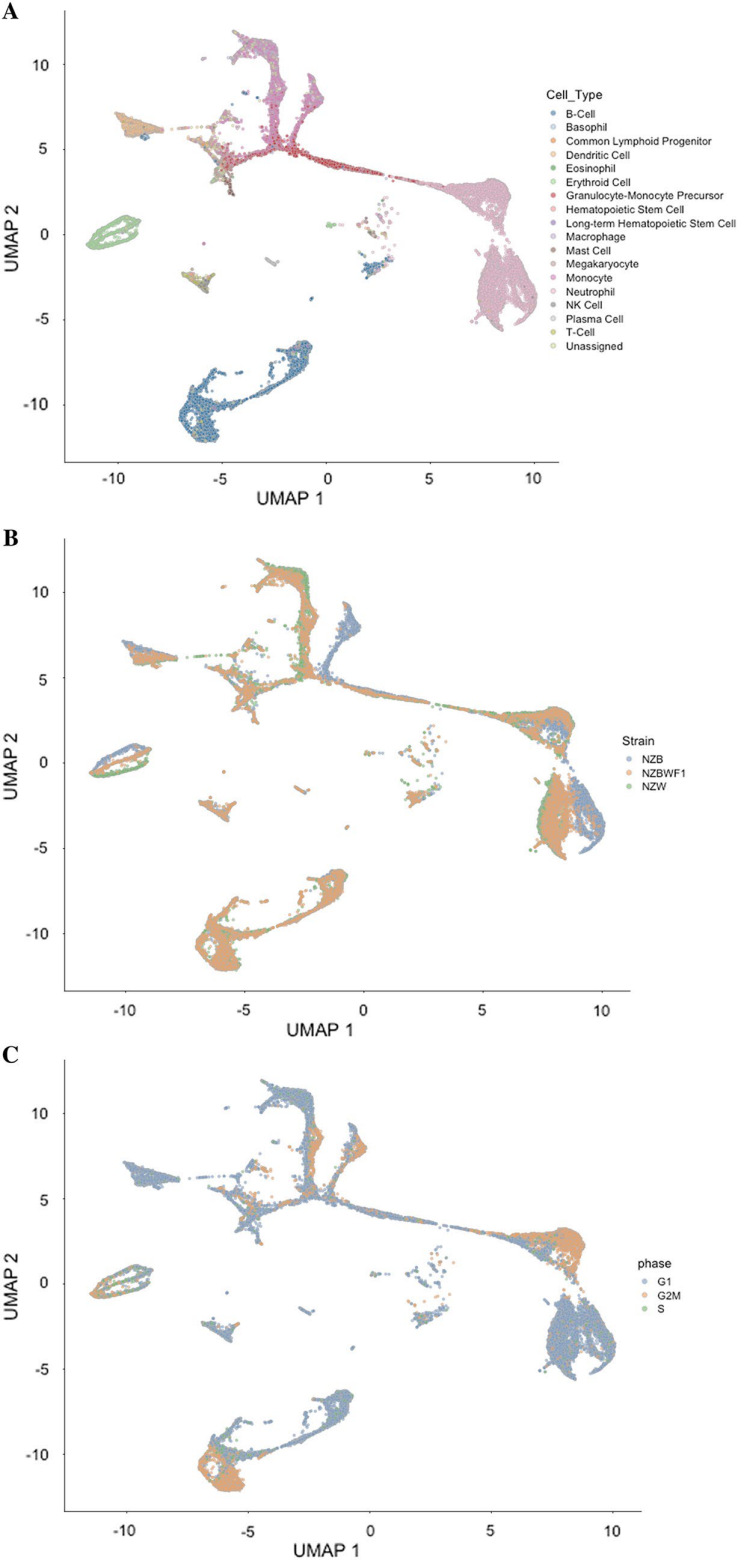
Dimensionality reduction of bone marrow cell gene expression. All plots are of the Uniform Manifold Approximation and Projection (UMAP). (**A**) Colored by assigned cell type. All three major hematopoietic lineages are present. (**B**) Colored by strain of origin for each cell. Note how the NZBWF1 cells often fall in between the NZW and NZB strains. (**C**) Colored by phase of the cell cycle. There is no significant difference in cell cycle phases between different strains.

A high cell viability was obtained using the aforementioned isolation method, as NZB, NZW, and NZBWF1 mice had an average of 88, 94, and 94 percent viability, respectively. After initial quality control, 31,053 cells remained for downstream analysis. All three major hematopoietic lineages (immune, erythroid, and megakaryocytic) were represented across each individual strain (Fig. 1A,B). Interestingly, the gene expression of some NZBWF1 cell types—for example, erythroid and neutrophils—fell precisely in between the two parent strains (Fig. 1B), suggesting that the phenotype of the NZBWF1 strain is contributed to equally by both parent strains. By assessing the expression of stereotypic cell cycle genes, the phase of the cell cycle was assigned to each cell in our dataset (Fig. 1C). As expected, intermediate cell types (e.g. granulocyte-monocyte progenitors) exhibited progression through the cell cycle. There was no significant difference in the number of cells in each phase when compared amongst different strains (data not shown).

**Figure 2:**
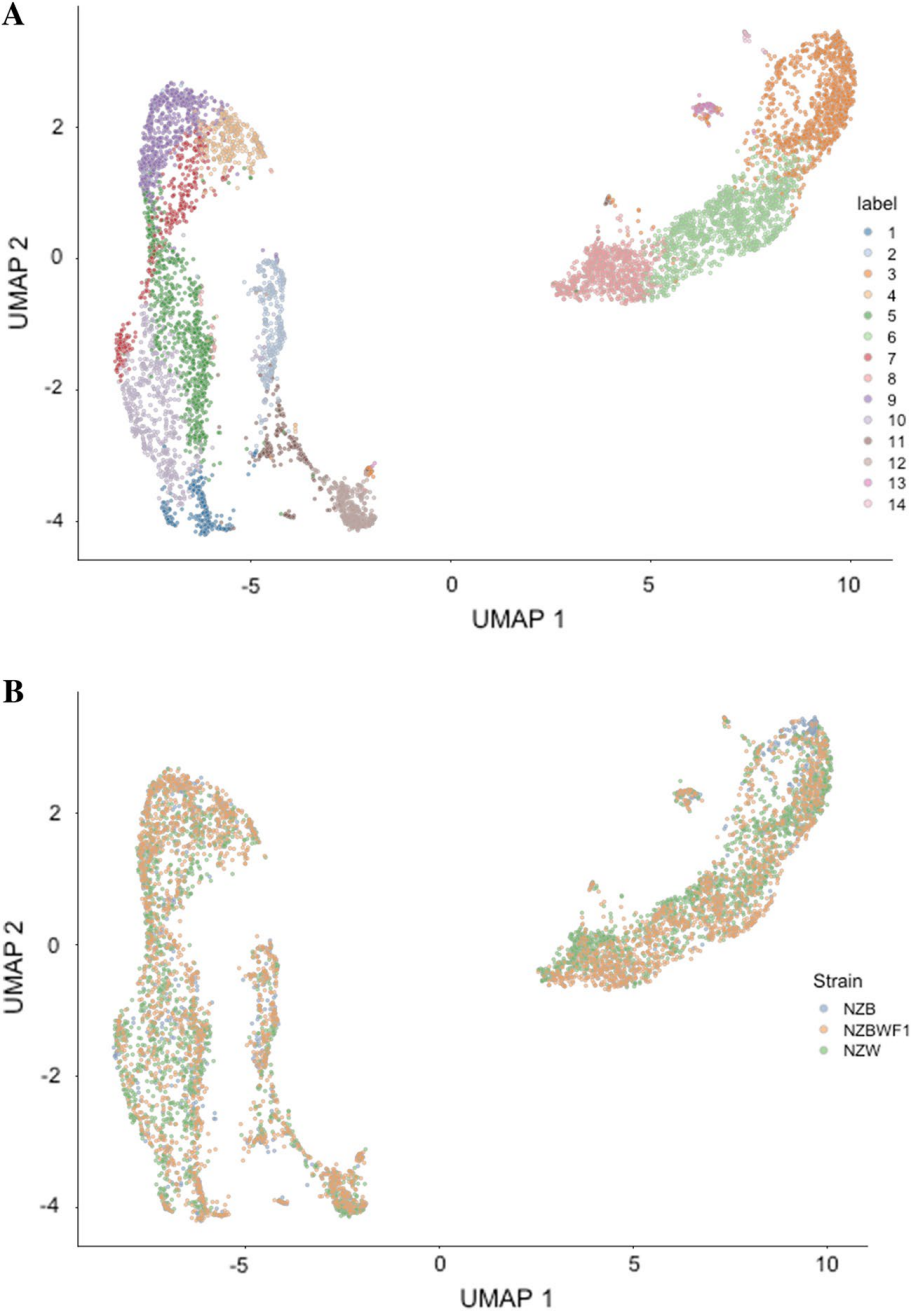
Dimensionality reduction of B-cells colored by cluster (**A**) and strain (**B**). There is no enrichment of any one cluster in the NZBWF1 strain as compared to the parent strains.

Next, the identification of any particular cell type exhibiting differential abundance in the NZBWF1 strain as compared to the NZB or NZW strain was examined. As shown in Table 1, B-cells and plasma cells from NZBWF1 mice exhibited an approximate onefold increase in abundance, supporting the key role of auto-antibody production in the SLE-like phenotype of this strain. Given this finding, a sub-cluster analysis of this B-cell population was conducted in order to discern whether any particular B-cell subtype is enriched in the NZBWF1 strain. Based on unsupervised clustering, there were 14 subtypes of B-cells present in the dataset (Fig. 2A). There was no significant difference in the abundance of any of these subtypes across strains (Fig. 2B). However, it is possible that the dataset may not be sufficiently powered to detect significant differences in these sub-populations. Similar to results for the entire dataset, NZBWF1 B-cells were not enriched in any given phase of the cell cycle when compared to the parent strains (data not shown).

**Table 1:** Differential abundance of cell types across strains.

Cell type	logFC	logCPM	F	P value
B-cell	0.98817355	17.6832439	17.9897336	0.00122998
Plasma cell	1.27773038	14.113683	11.0312179	0.00703153
Mast cell	− 0.4652928	12.7808536	3.31124615	0.0946657
T-cell	0.68143326	14.6009994	3.2124122	0.10125933
Neutrophil	− 0.3182438	18.3413162	2.56346925	0.13619568
Erythroid cell	− 0.5468718	15.7460808	2.25148264	0.1622982
Granulocyte-monocyte precursor	− 0.4410333	15.4648248	1.82167637	0.2048745
Hematopoietic stem cell	0.35080046	12.5860995	1.75501409	0.2107284
Macrophage	0.19599014	15.1284366	0.66806655	0.43017882
Monocyte	− 0.1525407	16.8989742	0.34377706	0.56914649
Megakaryocyte	− 0.1586588	14.3942908	0.24953066	0.62748041
Unassigned	0.08894057	15.0939569	0.19374589	0.6678987
NK cell	0.1427398	14.3660814	0.18558034	0.67513879
Dendritic cell	0.05500362	16.0549614	0.07041496	0.79538582

In order to assess gene expression, a pseudo-bulk analysis used to identify genes which were differentially expressed in the NZBWF1 as compared to NZB and NZW strains within each cell type. Overall, very few genes were differentially expressed (Table 2). For example, in B-cells only 14 genes were significantly upregulated and only 3 were downregulated. Thus, the unique auto-immune phenotype of the NZBWF1 strain may be driven by relatively small alterations in gene expression as compared to its parent strains. Given the relatively small number of genes differentially expressed, gene ontology and reactome analyses failed to identify any significant enrichment for gene ontology terms or reactome pathways, respectively.

**Table 2:** Number of differentially expressed genes across cell types.

	Downregulated	Not Significant	Upregulated	NA
B-cell	3	9674	14	21,362
Dendritic cell	2	6511	22	24,518
Erythroid cell	2	8421	25	22,605
Granulocyte-monocyte precursor	0	6900	14	24,139
Hematopoietic stem cell	0	770	2	30,281
Macrophage	0	5049	6	25,998
Mast cell	0	927	2	30,124
Megakaryocyte	0	4586	4	26,463
Monocyte	16	8886	45	22,106
Neutrophil	0	7930	24	23,099
NK cell	1	2567	5	28,480
Plasma cell	0	1410	5	29,638
T-cell	0	2024	3	29,026
Unassigned	1	5495	6	25,551

**Figure 3:**
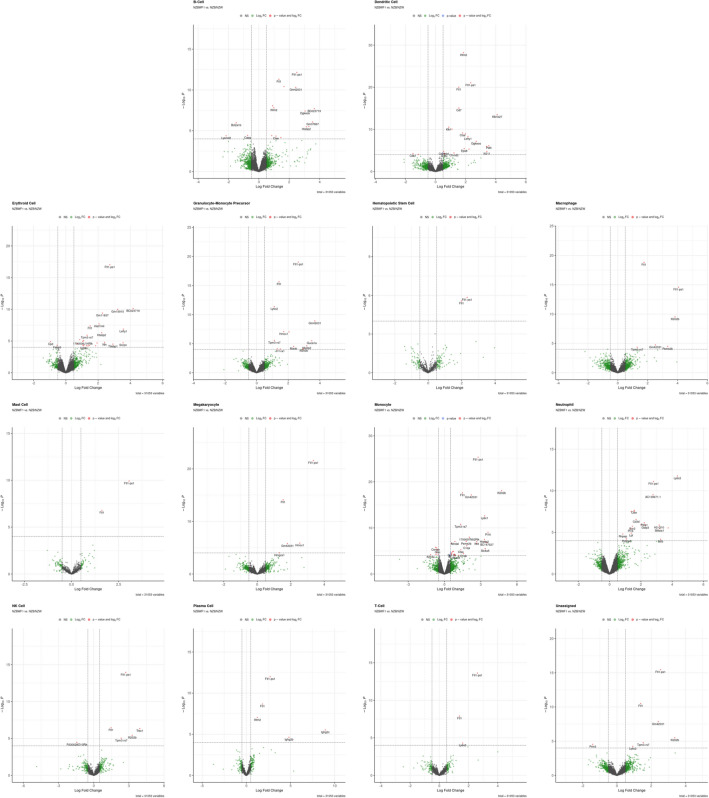
Volcano plots for each cell type.

The specific genes which were differentially expressed were remarkably consistent across all cell types (Fig. 3, Table 3, and Supplementary Table 2). Among the most significant genes was *Ftl1* and *Ftl1-ps1*, encoding ferritin light polypeptide 1 and ferritin light polypeptide 1 pseudogene 1, respectively. Additional genes which exhibited consistent upregulation across multiple cell types were *Ifitm2*, *Apobec3*, and *Ifi202b*, all genes which are regulated by the interferon pathway. *Ctse*, the gene encoding Cathepsin E, a protein involved in MHC class II antigen presentation, was also consistently upregulated.

**Table 3:** Top differentially expressed genes for each cell type as depicted in Fig. 3.

Cell type	Gene	logFC	P value	FDR
B-cell	Ftl1-ps1	2.50174162	7.07E−13	6.86E−09
	Ftl1	1.32126704	4.86E−12	2.35E−08
	Gm42031	2.4329193	4.81E−11	1.17E−07
	Tpm3-rs7	1.65835559	3.80E−11	1.17E−07
	Apobec3	0.91044418	8.94E−09	1.73E−05
Dendritic cell	Ifitm2	1.85651713	6.29E−29	4.11E−25
	Ftl1-ps1	2.33121597	8.69E−22	2.84E−18
	Ftl1	1.53974644	9.65E−21	2.10E−17
	Cd7	1.54503052	8.59E−16	1.40E−12
	Klk1b27	4.08987062	2.93E−14	3.83E−11
Erythroid cell	Ftl1-ps1	2.72595882	9.11E−18	7.69E−14
	Gm15915	3.21054274	9.91E−11	2.79E−07
	BC023719	4.17275982	7.45E−11	2.79E−07
	Gm11837	2.26635837	3.85E−10	8.14E−07
	Hist1h4i	2.06363105	1.95E−08	3.29E−05
Granulocyte-monocyte precursor	Ftl1-ps1	2.63673211	9.33E−20	6.45E−16
	Ftl1	1.41551313	2.22E−16	7.67E−13
	Ly6c2	1.11070042	4.23E−12	9.74E−09
	Gm42031	3.68043299	1.26E−09	2.17E−06
	Hmox1	1.70928675	8.60E−08	0.00011017
Hematopoietic stem cell	Ftl1	1.98368353	2.70E−06	0.00104082
	Ftl1-ps1	2.32695056	1.45E−06	0.00104082
Macrophage	Ftl1	1.75021657	1.53E−19	7.74E−16
	Ftl1-ps1	4.06719941	2.59E−15	6.56E−12
	Ifi202b	3.83046466	3.01E−10	5.07E−07
	Gm42031	2.52142643	1.81E−05	0.02281615
	Psme2b	3.3391237	3.31E−05	0.03350592
Mast cell	Ftl1-ps1	3.05990462	1.22E−10	1.13E−07
	Ftl1	1.6133699	1.65E−07	7.66E−05
Megakaryocyte	Ftl1-ps1	3.354989	2.69E−22	1.23E−18
	Ftl1	1.54781558	7.85E−15	1.80E−11
	Hmox1	2.53794703	1.33E−06	0.00189498
	Gm42031	1.8046175	1.65E−06	0.00189498
Monocyte	Ftl1-ps1	2.78859068	4.67E−26	4.18E−22
	Ifi202b	4.72648063	8.41E−19	3.76E−15
	Ftl1	1.48626521	2.47E−18	7.37E−15
	Gm42031	2.22773198	7.12E−18	1.59E−14
	Ly6c1	3.32351574	2.51E−13	4.49E−10
Neutrophil	Ly6c2	4.33353168	1.60E−12	1.27E−08
	Ftl1-ps1	2.80701994	7.69E−12	3.06E−08
	AC139671.1	2.77693183	3.03E−10	8.02E−07
	Ctse	1.56917145	2.23E−08	3.99E−05
	Ftl1	1.5759339	2.90E−08	3.99E−05
NK cell	Ftl1-ps1	2.73386052	1.11E−14	2.85E−11
	Trbc1	3.92664566	4.52E−07	0.00038791
	Ftl1	1.47947574	3.58E−07	0.00038791
	Ifi202b	3.2806935	3.72E−06	0.00239273
	Tpm3-rs7	2.34843827	9.22E−06	0.00474277
Plasma cell	Ftl1-ps1	2.6977062	9.79E−13	1.39E−09
	Ftl1	1.82543793	2.12E−09	1.50E−06
	Ifitm2	1.24298817	9.23E−08	4.35E−05
	Ighg2c	8.93073673	2.95E−06	0.00104291
	Ighg2b	4.82008471	2.36E−05	0.00666655
T-cell	Ftl1-ps1	2.60830889	2.32E−14	4.70E−11
	Ftl1	1.37918593	1.27E−08	1.29E−05
	Ly6c2	1.59713348	4.96E−05	0.03351994

The top 5 most significant genes are listed. For cell types with less than 5 significant genes, only those genes of statistical significance are shown.

Several genes of cryptic functional significance were similarly upregulated including *Tpm3-rs7*, *Htatip2*, and *Gm42031*. *Tpm3-rs7* (tropomyosin-related sequence 7) is a heretofore uncharacterized protein with putative actin filament binding. *Gm42031* is an uncharacterized locus (postulated to be a lncRNA) whose downregulation in macrophages has been previously linked to neuroinflammation^32^. However, the dataset of this highly inflammation-prone strain shows that it is upregulated. *Htatip2* is an oxidoreductase with a role in nuclear import signaling. There were no genes which exhibited consistent downregulation across all cell types.

Some genes were selectively altered in only specific cell types. For example, *Ly6c2* expression was upregulated across myeloid cell types. Monocytes over-expressed several complement genes namely, *C1qa* and *C1qb*. Dendritic cells over-expressed *Klk1* (kallikrein). As expected, there were very few T cells in the bone marrow; thus, the lymphoid lineage was unable to be assessed as a whole. Regarding B-cells, *Bcl2a1b*, an anti-apoptotic protein, was significantly downregulated in B-cells, suggesting that pro-survival signals may bypass immune checkpoints within the NZBWF1 mice. Plasma cells over-expressed *Ighg2b* and *Ighg2c*, further underscoring the central role of antibody production in the SLE-like phenotype.

## Discussion

SLE is a multifactorial syndrome which involves a complex interaction of genetic susceptibility and environmental factors/exposures. By conducting single cell RNA sequencing on the bone marrow of the SLE-prone NZBWF1 mouse strain and contrasting it to its two parent strains, neither of which develop overt autoimmunity to the degree of their progeny, this study sought to unravel immune factors which contribute to this syndrome. Importantly, this analysis was conducted prior to the development of the disease phenotype, allowing for the identification of potential predisposing factors which contribute to later autoimmunity.

First, the results showed enrichment of B cells and plasma cells in the bone marrow of NZBWF1 mice. These B cells expressed genes associated with activation and inflammation while the associated plasma cells exhibited upregulation of immunoglobulin G heavy chain. Taken together, these results underscore the central role of B cell activation and antibody production to the autoimmune phenotype of NZBWF1^33,34^. A prior single cell RNA sequencing study of human peripheral blood in SLE^35^ similarly identified alterations in B cells, noting enrichment of particular subpopulations of memory and activated B cells within the peripheral blood of SLE patients.

The interferon pathway is a well-known mediator of SLE in both mouse models^36^ and humans^37–39^. The finding of interferon activation further supports a key role for this pathway in the NZBWF1 and validates its use as a small animal model of SLE. The same study cited above^35^ also identified upregulation of the interferon pathway as of particular importance across a variety of cell types, including T-cells, monocytes, and dendritic cells in patients with SLE.

Of particular importance, our data showed marked upregulation of the ferritin light chain across all cell types in NZBWF1 mice relative to parental controls. Prior quantitative trait loci mapping identified a QTL associated with the SLE-like phenotype encompassing the *Ftl1* locus on chromosome 7^6,8^. Playing a central role in iron uptake and storage^40^, the ferritin light chain is also a well-known acute phase reactant with important immune signaling actions^41^. Indeed, the ferritin light chain has been shown to be the primary circulating form of ferritin and mediates many of its immune functions^42^. Canonically, monocytes are the primary source of ferritin light chains in this context. Indeed, in the setting of autoimmunity, high levels of circulating ferritin are associated with the macrophage activation syndrome^43,44^. Thus, the marked upregulation across all cell types in this dataset is notable and represents a potentially novel avenue of scientific and therapeutic investigation. Whether ferritin upregulation is a cause of underlying NZBWF1 autoimmune predisposition remains to be elucidated.

Several genes of enigmatic function were also identified, suggesting potentially interesting areas of further study. *Gm42031* was previously identified in a study of microglial activation. In that study, Wilson et al. identified its downregulation to be associated with neuro-inflammation^32^. Thus, the current finding of marked upregulation in the NZBWF1 strain in the context of inflammation is intriguing. Further characterization of *Gm42031* and its functional impact on immune regulation may be a novel area of investigation.

To our knowledge, this is the first study to leverage single cell RNA sequencing in examining the bone marrow of the NZBWF1 mouse strain. Prior studies in this model have examined single cell transcriptomics in the kidney and lung of affected mice later in the disease course^45,46^. There, the author’s identified extensive immune cell infiltration and activation, consisting of nearly all major immune cell types. Thus, our study uniquely examines the developmental milieu of these immune cells at an early time point, prior to the development of overt autoimmunity.

Our study has several limitations. First, we examined only a single time point. It is possible earlier time points would have identified more proximate genetic contributors to autoimmune predisposition in the NZBWF1 strain. Similarly, given this cross-sectional design, we cannot establish whether the observed gene expression changes cause autoimmunity. Future studies could examine longitudinal gene expression changes to more comprehensively chart the genetic and transcriptomic landscape of autoimmune development in the NZBWF1. Furthermore, targeted perturbations of the candidate pathways (e.g. ferritin light chain) could provide more insight into their causal role and establish therapeutic candidacy.

Taken together, the current data represent an atlas of hematopoietic phenotype early in the course of disease development of the NZBWF1 mouse. The data are not only consistent with the B cell activation and interferon signaling expected of SLE, but also identify several novel areas of interest, most notably, the ferritin light chain signaling axis. Data sets such as the one from this study may be useful for gaining a better understanding of this devastating disease leading to better therapeutic approaches.

## Supplementary Information

Supplementary Information 1.

Supplementary Table 1.

Supplementary Table 2.

Supplementary Figure 1A.

Supplementary Figure 1B.

Supplementary Figure 1C.

Supplementary Figure 2A.

Supplementary Figure 2B.

Supplementary Figure 2C.

## Data Availability

Raw data has been deposited in the NCBI-GEO under accession number GSE174728 (https://www.ncbi.nlm.nih.gov/geo/query/acc.cgi?acc=GSE174728). Code for data analysis is available in GitHub (https://github.com/styounes/SLE_scRNAseq).
