# Supplementary material for: Single cell RNA sequencing reveals ferritin as a key mediator of autoimmune pre-disposition in a mouse model of systemic lupus erythematosus
Source: Sci Rep. 2021 Dec 20;11:24245. doi: 10.1038/s41598-021-03649-2 (PMC8688484; doi:10.1038/s41598-021-03649-2)
Supplement: Supplementary file 1 — Supplementary Information 1. [file 41598_2021_3649_MOESM1_ESM.docx]

**Supplementary Figure and Table Legends**

**Supplementary Figure 1**. Quality control metrics. (A) Total number of UMI’s detected, number of unique genes detected, and the percentage of mitochondrial genes per cell for each cell from each mouse. The discard metric was applied to any cell not meeting one of any given quality control metric (see text for details). NZB = New Zealand Black, NZW = New Zealand White, SLE = New Zealand Black-White F1. (B) Total library size (sum) vs mitochondrial percentage. There are no cells with high library sizes (i.e. high quality cells) with similarly high mitochondrial gene content. This ensures that the mitochondrial percent cutoff does not inadvertently remove metabolically active cells. (C). Volcano plot depicting kept and discarded genes. The plot shows that no given gene group is enriched (each dot) in discarded cells.

**Supplementary Figure 2**. Subclustering analysis of B-Cells. (A) Graph of B-Cell subclusters. Each node is labeled with its respective cluster number. The width of the edges represents the weight (i.e. similarity) between each node. Overall, the edges are relatively thin, indicating good clustering results (i.e. dissimilarity between different clusters). (B) Subclustering re-assignment probability based on bootstrapping technique. The graph shows that each subcluster is remarkably stable (i.e. low probability of reassigning cells with displacement of gene counts). (C) Subclustering stability based on a modularity approach. Once again, clusters exhibit relative stability (low inter-cluster modularity scores).

**Supplementary Table 1.** List of genes used to assign cell types.

**Supplementary Table 2.**  Table of all differentially expressed genes in each cell type.
