# Supplementary figures and images for: Single cell RNA sequencing reveals ferritin as a key mediator of autoimmune pre-disposition in a mouse model of systemic lupus erythematosus
Source: Sci Rep. 2021 Dec 20;11:24245. doi: 10.1038/s41598-021-03649-2 (PMC8688484; doi:10.1038/s41598-021-03649-2)

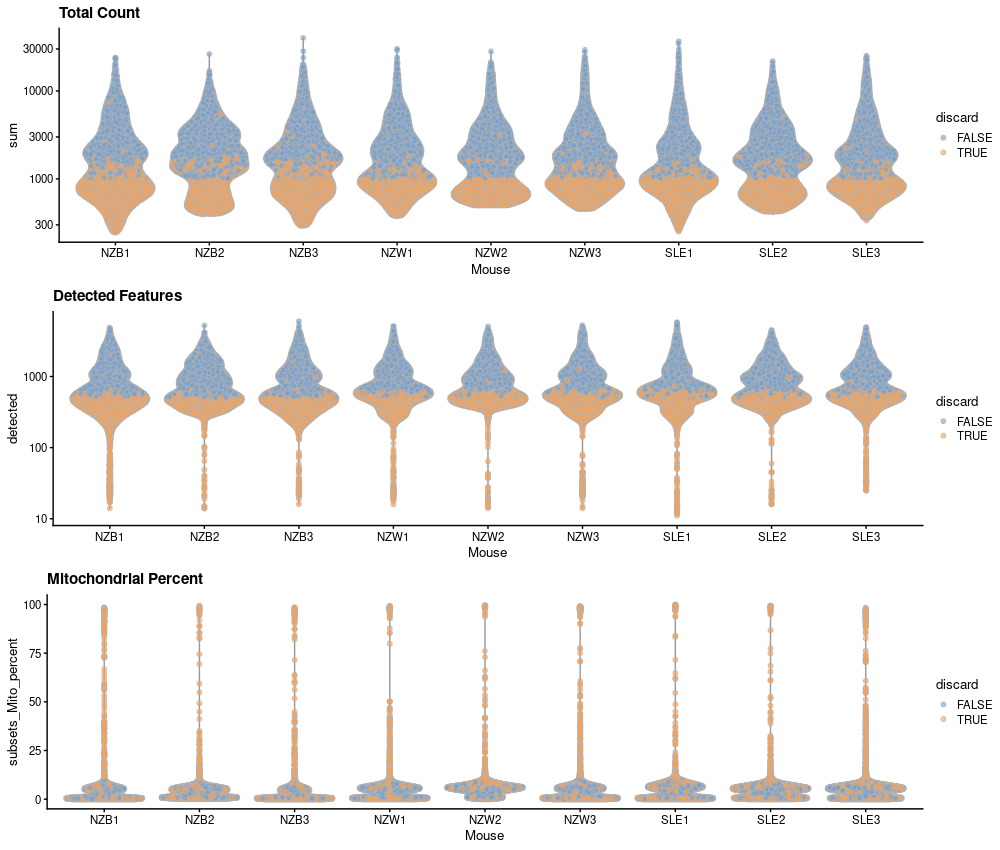

Supplement: Supplementary file 4 — Supplementary Figure 1A. [file 41598_2021_3649_MOESM4_ESM.tiff]

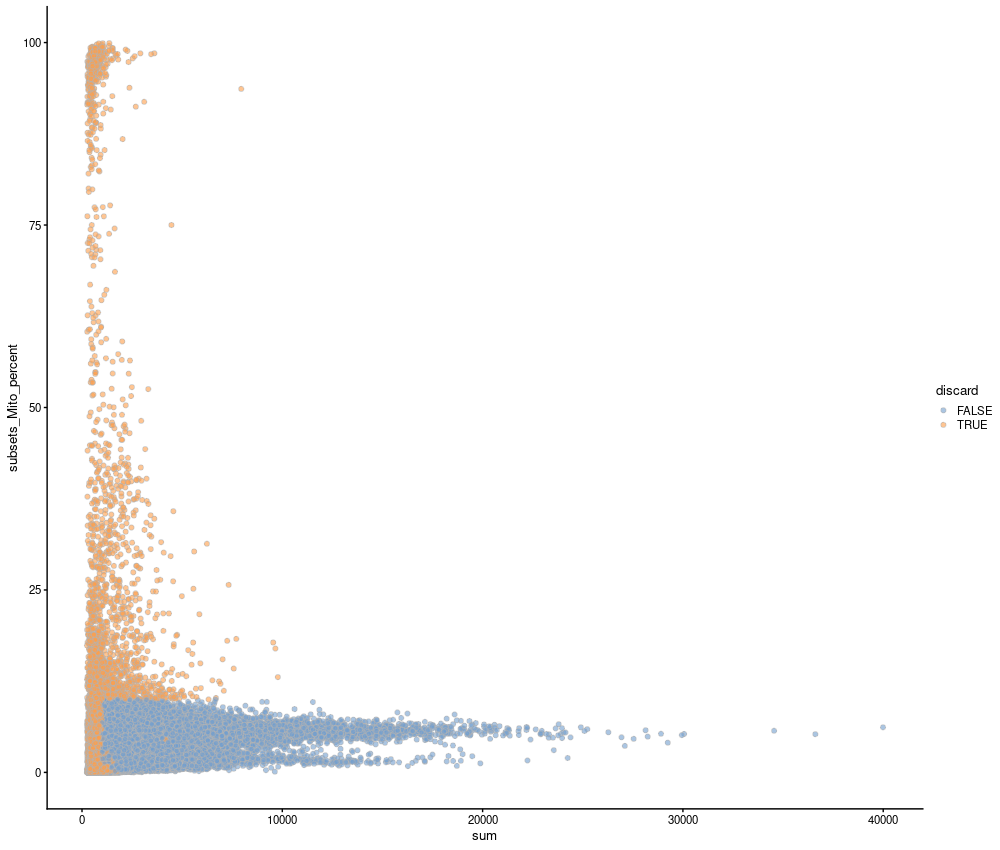

Supplement: Supplementary file 5 — Supplementary Figure 1B. [file 41598_2021_3649_MOESM5_ESM.tiff]

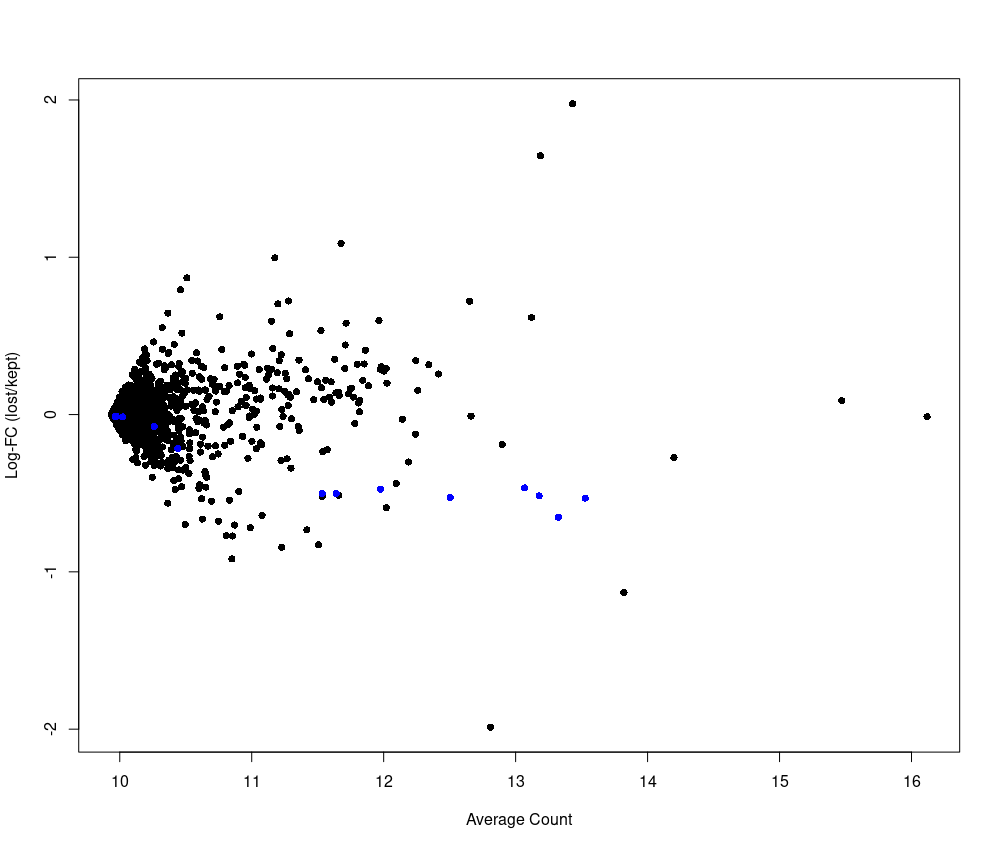

Supplement: Supplementary file 6 — Supplementary Figure 1C. [file 41598_2021_3649_MOESM6_ESM.tiff]

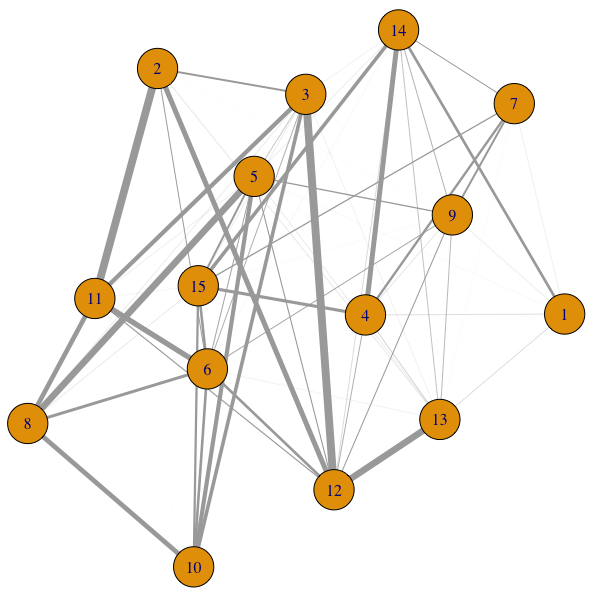

Supplement: Supplementary file 7 — Supplementary Figure 2A. [file 41598_2021_3649_MOESM7_ESM.tiff]

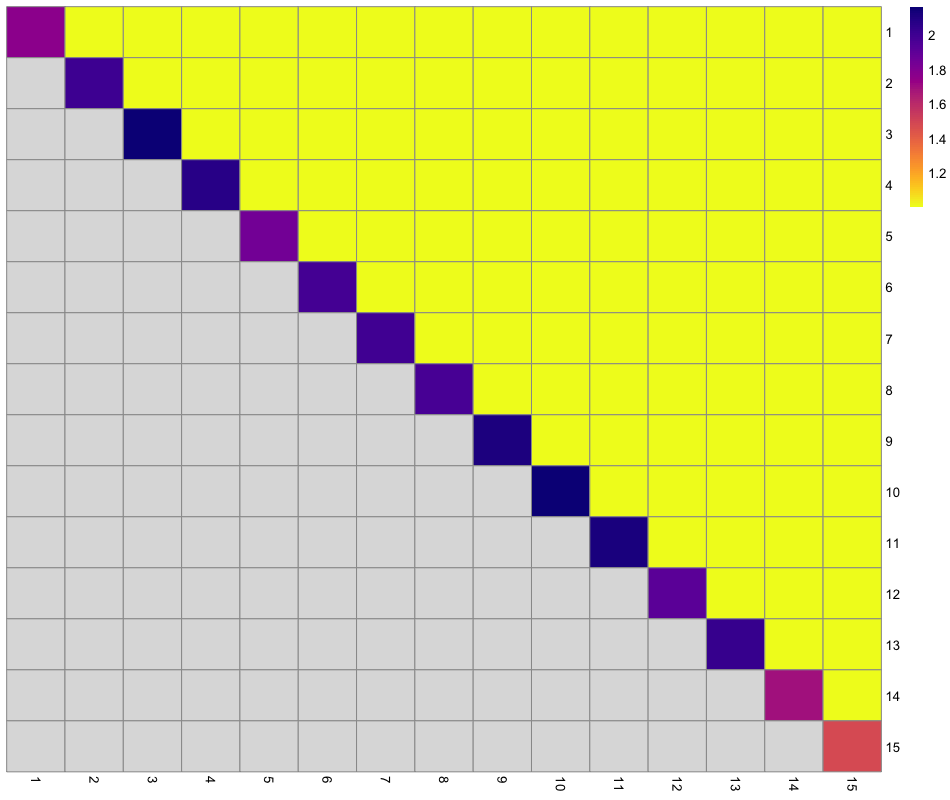

Supplement: Supplementary file 8 — Supplementary Figure 2B. [file 41598_2021_3649_MOESM8_ESM.tiff]

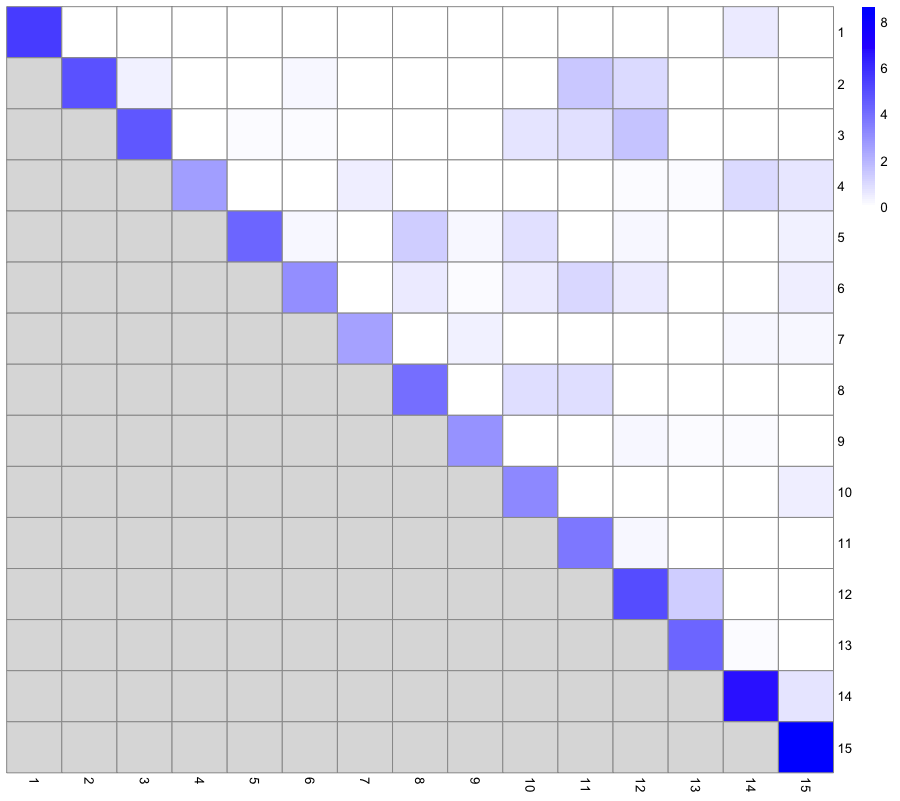

Supplement: Supplementary file 9 — Supplementary Figure 2C. [file 41598_2021_3649_MOESM9_ESM.tiff]
